# Editorial for the Special Issue “Genomics of Extremophiles and Archaea”

**DOI:** 10.3390/microorganisms14020433

**Published:** 2026-02-12

**Authors:** Shiladitya DasSarma, Sean P. Kennedy

**Affiliations:** 1 Department of Microbiology and Immunology, Institute of Marine and Environmental Technology, University of Maryland School of Medicine, Baltimore, MD 21202, USA; 2 Département de Biologie Computationnelle, Institut Pasteur, Université Paris Cité, F-75015 Paris, France; sean.kennedy@pasteur.fr

The number of sequenced extremophiles and archaea has exploded since the last decade of the 20th century [1,2,3]. Advances have been driven by the isolation of novel microorganisms from extreme environments and the increasingly powerful sequencing and analysis methods available. The rich biology of extremophilic microorganisms is revealing mechanisms of tolerance and adaptation to extremes, their community structure and dynamics, and providing clues to the origin and evolution of life on Earth. All of these areas were covered in the Special Issue entitled “Genomics of Extremophiles and Archaea”.

Among the most extreme environments on Earth is Mount Everest, where two novel radiation-resistant *Sphingomonas* species were isolated and studied by Liu et al. (Contribution 1). The genomic analyses of these bacteria showed that multiple radiation protection and DNA damage repair mechanisms are present, as well as genes encoding enzymes that detoxify reactive oxidative species and support the synthesis of antioxidative pigments and carotenoids. Related to radiation resistance, Nag et al. (Contribution 2) combined the physiological and genomic analysis of a diverse group of halophilic Archaea isolated from high-elevation salt flats, warm and cold hypersaline surface lakes near sea level, and subsurface halite deposits from the ancient Permian period. Surface environments exposed to high solar irradiance were found to be the most UV-tolerant, while subsurface species were the least tolerant. The latter were correlated with the absence of DNA photolyase genes and with deviations in amino acid residues at key positions in the encoded enzyme, providing insights into their natural UV tolerance and survival in ancient halite deposits, a potential analog of Mars. In another study of a hypersaline environment in Guerrero Negro, Mexico, García-Maldonado et al. (Contribution 3) examined methanogenic archaeal communities in microbial mats. Phylogenetic environmental clusters of methanogenic archaea were observed and based on the detection of specific genes in the metagenome, all four methanogenic pathways were hypothesized to occur in these microbial mats, including contributions from low-abundance methanogens. An extensive review of the literature on archaeal restriction–modification systems, which have expanded rapidly with the advent of PacBio SMRT sequencing, was published by Anton and Roberts (Contribution 4). Their survey of the New England Biolabs open database of restriction enzymes, REBASE, utilizing over 500 completely sequenced archaeal genomes, revealed the target recognition sites of DNA methyltransferases and they found organizational structures and protein sequences that were highly similar to those of bacteria, suggesting that both groups acquired systems from a shared genetic pool through horizontal gene transfer.

Two studies were published that focused on marine microbes, one by Li et al. (Contribution 5) on a deep-sea *Shewanella* isolate and another by Patwardhan et al. (Contribution 6) on a *Varunaivibrio sulfuroxidans* strain, an α-proteobacterium from shallow waters in the Tyrrhenian Sea off the coast of Italy. The genome of *V. sulfuroxidans* displayed metabolic versatility for adaptation to the dynamic environmental conditions of sulfidic gas vents and a mixotrophic lifestyle. Interestingly, the bacterium encodes a variety of genes involved in sulfur metabolism as well as nitrate and oxygen respiration and carbon fixation, in addition to glycolysis and the TCA cycle. The *Shewanella* species was isolated from the Mariana Trench at a depth of 5900 m and found to harbor one of the largest genomes in this genus. A comprehensive genomic analysis established metabolic versatility related to adaptation to its oligotrophic environment. Finally, Wang et al. (Contribution 7) characterized the growth and metabolism of *Acidithiobacillus ferrooxidans* under electroautotrophic and chemoautotrophic conditions. Differential expression analysis was used to analyze the two growth conditions and provide a model for electroautotrophic growth through the use of extracellular electrons.

This Special Issue provides a fascinating cross-section of genomic studies in the field of extreme microorganisms, as well as directions for future work that are likely to open new opportunities to address questions in both fundamental and applied research.
